# Preparation and Synergistic Effect of Biomimetic Poly(lactic acid)/Graphene Oxide Composite Scaffolds Loaded with Dual Drugs

**DOI:** 10.3390/polym14245348

**Published:** 2022-12-07

**Authors:** Shuqiong Liu, Wanzhu Li, Zhenyi Xu, Jiapeng Hu, Fangfang Wu, Yuying Zheng

**Affiliations:** 1 College of Ecology and Resource Engineering, Wuyi University, Wuyishan 354300, China; 2 College of Materials Science and Engineering, Fuzhou University, Fuzhou 350108, China

**Keywords:** salvianolic acid B, aspirin, dual drug-loaded, biomimetic composite scaffolds

## Abstract

To promote the bone repair ability of drug-loaded scaffolds, poly(lactic acid) (PLA)/graphene oxide (GO)/Salvianolic acid B (Sal-B)/aspirin (ASA) dual drug-loaded biomimetic composite scaffolds were prepared. The results showed that the addition of these two drugs delayed the gel formation of the composite system, but a biomimetic nanofiber structure could still be obtained by extending the gel time. The addition of Sal-B increased the hydrophilicity of the scaffold, while an increase in ASA reduced the porosity. Dual drug-loaded scaffolds had good haemocompatibility and synergically promoted the proliferation of MC3T3-E1 cells and enhanced alkaline phosphatase activity. Sustained-release experiments of the two drugs showed that the presence of ASA slowed the cumulative release of Sal-B, while Sal-B promoted the release of ASA. Kinetic modeling showed that the release of both drugs conforms to the Korsmeyer–Peppas model, but Sal-B conforms to the Fick diffusion mechanism and ASA follows Fick diffusion and carrier swelling/dissolution.

## 1. Introduction

Orthopedic diseases have become some of the most widespread diseases. Bone grafting can be utilized to treat these orthopedic diseases, so bone graft materials are a key factor in the treatment of these diseases. Bone healing and regeneration are driven by a variety of growth factors. Numerous studies have reported that the continuous release of growth factors promotes cell proliferation in clinical fracture healing experiments [1]. Additionally, local administration of growth factors has a synergistic effect (i.e., enhances bone formation) on osteoprogenitor cells and osteoblasts both in vitro and in vivo [2,3]. Studies have increasingly been interested in site-specific spatial and temporal encapsulation and the release of a wide range of bioactive molecules. Graded continuous release of multiple drugs significantly improves tissue regeneration compared to the controlled release of a single biological signal. Recently, studies reported the enhancement of pluripotent cell differentiation, alkaline phosphatase (ALP) activity and matrix mineralization through the continuous delivery of BMP-2 and IGF-I via a novel layered biodegradable polymer scaffold [4]. Topical antibiotics and the timely control of growth factor release may help prevent infection and strengthen bone healing. To this end, Strobel et al. developed a variable-order drug delivery system with three different release modes: (ⅰ) gentamicin is released; (ⅱ) IGF-I is released explosively and then continuously; and (ⅲ) slow and sustained release of BMP-2. BMP-2 stimulated the metabolic activity and ALP activity of C2C12 cells after 2 weeks, while the continuous release of IGF-I stimulated cell proliferation [5]. The sequential release of these three drugs had a superposition effect on tissue repair. Çakır-Özkan et al. prepared a PLLA-PEG tissue-engineered scaffold with recombinant human bone morphogenetic protein (rhBMP-2) and vascular endothelial growth factor (rhVEGF165) that maintained different release rates of these two growth factors [6]. The results showed that the rhBMP-2-containing group had the largest bone volume, and the VEGF165-containing group had the largest vessel volume, but scaffolds containing both rhBMP-2 and VEGF165 provided the best results while increasing new bone remodeling. An et al. successfully constructed gelatin/PLGA nanofiber scaffolds loaded with VEGF and BMP-2 and found that the two growth factors could be released sequentially, during which VEGF and BMP-2 promoted the adhesion, proliferation and differentiation of bone marrow mesenchymal stem cells (BMSCs) [1].

Additionally, previous studies have shown that combining two drugs that both promote bone repair can have an even greater combined effect than simply an additive effect of the two drugs. For example, Wang et al. showed that salvia miltiorrhiza combined with dexamethasone increased the osteogenic conversion rate and the differentiation and proliferation of osteoblasts [7]. Yang et al. showed that salvia miltiorrhiza combined with BMP-2 promoted the differentiation of rat BMSCs into cardiomyocytes [8]. Shoba et al. found that salvianolic acid B (Sal-B) and L-magnesium ascorbate 2 phosphate jointly promoted the differentiation of myoblasts with good cell morphology [9]. Xu et al. also showed that loading EPO and ASA into a CS/β-GP/gelatin hydrogel at the same time provided anti-inflammatory and periodontal tissue regeneration effects [10]. Yao et al. showed that the combination of salvia miltiorrhiza and aspirin (ASA) had a better antiplatelet aggregation effect than their use alone [11].

ASA is a non-steroidal anti-inflammatory drug with analgesic, antipyretic, anti-inflammatory, anti-rheumatic, and anti-thrombotic effects, so it plays an important role in many biomedical fields in the treatment of diseases [12,13]. Studies have shown that ASA has effects on bone repair and bone therapy; for example, ASA inhibits the differentiation and maturation of osteoclasts and significantly promotes bone repair in rodents [14]. Ren et al. reported that compared with pure titanium implants, ASA-loaded implants significantly promoted the proliferation and differentiation rate of MC3T3-E1 cells into osteoblasts [12]. Other studies have also shown that ASA impacts bone metabolism and health, promoting the osteogenic differentiation of BMSCs, inhibiting the adipogenic differentiation of BMSCs, activating osteoblasts and inhibiting osteoclasts [12,15,16,17,18]. At the same time, studies have shown that ASA promotes bone formation by inhibiting the expression of inflammatory factors such as IFN-γ and TNF-α, improving the bone marrow microenvironment and enhancing the immune regulation of BMSCs [14,19]. A study by Tang and Li showed that ASA accelerates bone repair, possibly by controlling the balance between bone formation and resorption and preventing osteoclast differentiation and maturation [19,20]. Furthermore, ASA has been reported to prevent soft tissue invasion into bone defects during tissue engineering surgery [21].

Sal-B is a water-soluble phenolic acid extracted from the root or rhizome of salvia miltiorrhiza, a traditional Chinese medicine, and it has been shown to have a positive therapeutic effect on cardiovascular diseases [22,23]. In addition to its beneficial effects in cardiovascular diseases, Sal-B has also been studied in bone repair and reconstruction. Studies by Yan et al. showed that salvia miltiorrhiza activates blood circulation and reduces stasis, channeling the meridians and activating the collaterals, and also impacts bone cells, which can stimulate osteogenesis and promote bone healing [24]. Cui et al. showed that salvia miltiorrhiza extract promoted the secretion of osteocalcin and ALP, thus treating osteoporosis in rats [25]. Moreover, that study also showed that salvia miltiorrhiza extract inhibited the formation of adipocytes but promoted osteoblast differentiation. He et al. showed that Sal-B accelerated early fracture healing and could be used as a treatment option for patients with traumatic fractures [26]. Fan et al. showed that it was also effective in orthopedic repair and improved the growth and mineralization of osteoblasts [27]. Additionally, studies have shown that Sal-B inhibits bone loss, promotes vascularization, stimulates cell metabolism, and enhances cell differentiation and proliferation, accelerating bone healing [28]. Other studies have demonstrated that Sal-B stimulates the total metabolic activity and ALP activity of osteoblasts through the ERK signaling pathway [29]. Therefore, Sal-B has potential value in inducing bone regeneration in the clinical application of bone tissue engineering.

We have successfully prepared PLA/GO/ASA and PLA/GO/Sal-B drug-loaded biomimetic composite scaffolds, and the scaffolds have good slow-release performance for ASA and Sal-B, respectively. In order to increase the bone inductance of the scaffold, hydroxyapatite was added to prepare a PLA/HA/GO/ASA ternary drug-loaded composite scaffold [30]. The addition of hydroxyapatite promotes the ability to induce apatite formation. According to previous studies, a PLA/GO composite scaffold is a very effective drug loading matrix. In order to explore the synergistic effect between different drugs, we co-loaded Sal-B and ASA onto the PLA/GO composite biomimetic scaffold prepared in this paper, expecting to obtain a better drug-loading scaffold, and we studied the effects of the synergistic effect between the two drugs on drug release performance and other properties. GO plays an important role in the performance of the PLA/GO scaffold. GO has been widely used in the field of biomedicine because of its unique physical properties, particularly in drug delivery [31,32]. In fact, GO has a large number of hydrophilic functional groups and a certain π-conjugated structure, and these characteristics endow it with strong amphiphilicity and enhance its easy dispersion in most polymers [33]. This amphiphilicity enables GO to disperse on and cover the PLA nanofibers uniformly, which is particularly important for improving the hydrophilicity of the drug-loaded scaffolds. At the same time, a previous study showed that GO and ASA or Sal-B can form π–π stacking interactions [12]; hence, the good dispersion of GO in PLA can affect the distribution of ASA and Sal-B in the carrier and further affect the release of drug. However, an investigation of the combination of ASA, a Western medicine, and Sal-B, a traditional Chinese medicine, has not been reported in bone tissue engineering. Therefore, we assessed the combination of these drugs using poly(lactic acid) (PLA)/graphene oxide (GO) with a biomimetic extracellular matrix structure as a carrier. The structural morphology, physical and chemical properties, water absorption, porosity, blood compatibility, cytotoxicity, ALP activity and sustained-release performance of bionic scaffolds loaded with different concentrations of Sal-B and ASA were investigated, providing a theoretical basis for the clinical application of bionic scaffolds combining Chinese and Western drugs.

## 2. Materials and Methods

### 2.1. Materials

PLA (3052D; Mn, 250,000 g/mol) was purchased from USA NatureWorks Co., Ltd. (Plymouth, MN, USA), Aspirin (ASA, AR) was supplied by Aladdin Reagent Co., Ltd. (Shanghai, China), Salvianolic acid (Sal-B) was purchased from Jiangsu Yongjian Pharmaceutical Technology Co., Ltd. (Taizhou, China). GO was prepared in our laboratory as described previously [34]. All other reagents and solvents used in this work were of analytical-reagent grade and purchased from Sinopharm Chemical Reagent Co., Ltd., Shanghai, China.

### 2.2. Fabrication of Dual Drug-Loaded Biomimetic Composite Scaffolds

**Figure 1:**
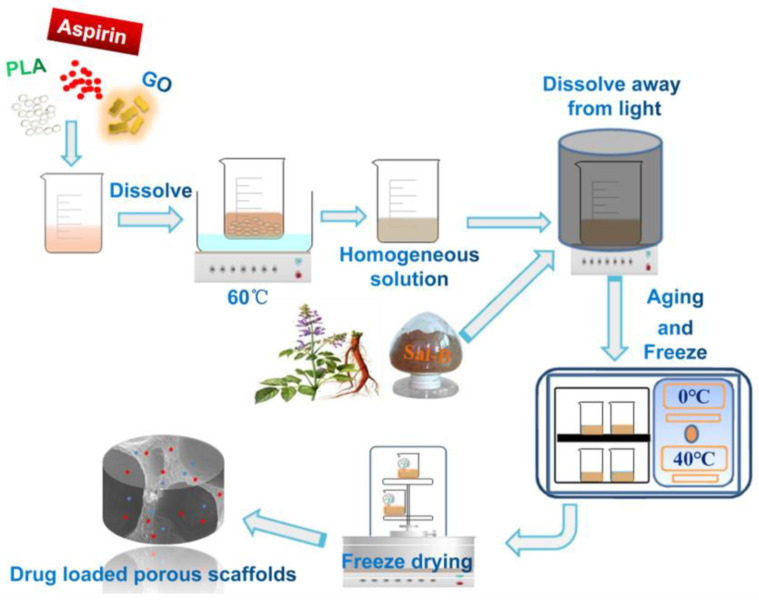
The flow chart of fabrication of dual drug-loaded biomimetic composite scaffolds.

GO (fixed at 2.0 wt% of GO relative to PLA) and ASA were ultrasonically dispersed in a dioxane/water mixture, and PLA (10.0 wt%; *w*/*w*) was successively added to the mixed solution and dissolved with stirring at 60 °C until a homogeneous mixed solution was obtained. Then, a corresponding amount of Sal-B was added and stirred in the dark for another 20 min to form a homogeneous solution. The solution was poured into the mold, rested at 0 °C overnight in the dark until the solution formed a gel and was then frozen at −40 °C for 3 h. Finally, the scaffold was freeze-dried in a vacuum (−54 °C) for 3 d and stored in a dry place away from light. When investigating variable levels of Sal-B, the amount of ASA was fixed at 5.0 wt% relative to PLA, while the amount of Sal-B was 0, 1.0, 3.0 and 5.0 wt% relative to PLA, with these groups named S0A5, S1A5, S3A5, and S5A5, respectively. When variable levels of ASA were assessed, the content of Sal-B was fixed at 3.0 wt%, and the amount of ASA was increased by 10.0 wt% and 15.0 wt% (the content was still relative to the quality of PLA). These groups were named S3A10 and S3A15, respectively. Table 1 presents the composition ratio of composite scaffolds with different drug contents. Figure 1 shows the flow chart of fabrication of dual drug-loaded biomimetic composite scaffolds.

### 2.3. Characterization of PLA/GO/ASA/Sal-B Dual Drug-Loaded Biomimetic Composite Scaffold

The morphology of PLA/GO/ASA/Sal-B dual drug-loaded biomimetic composite scaffolds was observed by scanning electron microscopy (SEM) (Vega 3 SBH, TESCAN CO., Ltd., Brno, Czech Republi) before and after drug release and platelet adhesion. Moreover, the influence of different ASA and Sal-B contents on the overall crystallinity of the dual drug-loaded biomimetic composite scaffold was analyzed by X-ray diffraction (XRD) (D8 Advance, Bruker Ltd., Saarbrucken, Germany). Furthermore, functional groups of the PLA/GO/ASA/Sal-B dual drug-loaded biomimetic composite scaffolds were confirmed using the Fourier-transform infrared spectroscopy (FTIR) spectra (Nicolet IS5, Thermo Fisher Ltd., Madison, WI, USA). The spectra were collected in transmission mode over the wavenumber range of 4000–400 cm^−1^.

### 2.4. Water Absorption and Porosity of the PLA/GO/ASA/Sal-B Dual Drug-Loaded Biomimetic Composite Scaffold

For the water absorption test, different samples were dried at 35 °C for 24 h. After weighing (*W*_1_), the specimens were soaked in distilled water for 24 h. The samples were then retrieved, dried with filter paper, and reweighed (*W*_2_). The water absorption of PLA/GO/ASA/Sal-B dual drug-loaded biomimetic composite scaffolds was calculated using Equation (1)

(1) $$W(\%)=\frac{W_{2}-W_{1}}{W_{1}}\times 100\%$$

where *W*% represents the water absorption of the prepared scaffold, *W*_1_ is the initial mass of the sample, and *W*_2_ is the mass of the sample 24 h after water uptake [34]. Three parallel samples were tested for each specimen.

The porosity of the PLA/GO/ASA/Sal-B dual drug-loaded biomimetic composite scaffolds was determined by a simple method described in a previous work [35].

### 2.5. Cell Culture

The biocompatibility of the dual drug-loaded biomimetic composite scaffolds was studied using a mouse pre-osteoblast cell line, MC3T3-E1 (Saibai Kang Biology, Shanghai, China). The cells were propagated in a conventional growth medium and incubated in a humidified atmosphere of 5% CO_2_ at 37 °C [36]. The medium was replaced with a fresh one every 3 days.

### 2.6. Cell Proliferation

The samples were cut into small pieces (1 × 10 × 2 mm^3^) and UV disinfected for 24 h. The MC3T3-E1 cells were seeded in 24-well plates (*n* = 3) at a seeding density of 10^4^ cells/well, cultured with dual drug-loaded biomimetic composite scaffolds containing different Sal-B and ASA contents and incubated in a standard incubator (37 °C and 5% CO_2_). Cell proliferation was investigated using Cell Counting Kit-8 (CCK-8, HY-K0301, MCE, Monmouth Junction, NJ, USA). After the cells were cultured for 1, 3 and 5 days, the cell-seeded scaffolds were rinsed with phosphate-buffered saline (PBS) twice and incubated with 500 μL of CCK-8 solution (10%) for another 2 h at 37 °C and 5% CO_2_. The formazan solution was transferred to a 96-well plate after incubation, and the optical density (OD) was measured at 450 nm using a microplate reader (EPOCH2, Biotek, Winooski, VT, USA). The obtained value was proportional to the number of adhered cells in each well.

### 2.7. Alkaline Phosphatase (ALP) Activity

The osteogenic differentiation of MC3T3-E1 cells was evaluated by measuring their ALP activity. MC3T3-E1 cells were inoculated in six-well plates at a density of 1.0 × 10^4^ cells/well and cultured adherent in a 5% CO_2_, 37 °C constant-temperature incubator. The scaffolds with different Sal-B and ASA contents were then added into each group. The cells were collected at 3, 7 and 14 days, and the ALP expression was detected following the instructions of the ALP activity detection kit. For cell lysate preparation, the culture medium was removed. The cells were washed with PBS, digested with trypsin, and we added 200 μL of protein lysate. The lysate was fully blown and centrifuged at 4 °C, 10,000× *g* for 10 min. The supernatant was obtained for detection. Afterward, 50 μL of the sample was added to the well of the ELISA plate, which was then vibrated for 30 s and incubated at 37 °C for 30 min. The reaction was stopped by adding 100 μL of stop solution to the well. The sample was thoroughly mixed, and the OD value was measured at 405 nm using a microplate tester.

### 2.8. In Vitro Release of ASA and Sal-B

Standard solutions of Sal-B and ASA were tested for absorbance at 270 nm and 286 nm, measured with a UV-visible spectrophotometer (UV-1100, Shanghai Meipuda Instruments Co., Ltd., Shanghai, China), and corresponding standard curves were made. The molar absorbance coefficients of ASA and Sal-B at 270 nm and 286 nm were obtained according to the equation of the standard curve. According to the absorbance, the concentration of the two drugs in the mixed solution was calculated. The drug release process of dual drug-loading scaffolds was measured by immersing a pre-weighed scaffold (approximately 0.03 g) in 25 mL PBS solution under a constant temperature while stirring (150 rpm; 37 °C). An aliquot (3 mL) of the solution from the release medium was removed at pre-determined time points and replaced with fresh buffer (3 mL). ASA and Sal-B absorbance in the solution were measured at 270 nm and 286 nm, respectively, and the cumulative release percentage (*η*) was calculated as follows:

(2) $$\eta =m_{n}\times 100\%/M$$

where m_n_ is the amount of ASA released from the PLA/GO/Sal-B/ASA composite scaffold at time t, and *M* is the total mass of ASA preloaded on the PLA/HA/GO/ASA composite scaffold.

### 2.9. Haemocompatibility

The haemocompatibility of the PLA/GO/Sal-B/ASA dual drug-loaded biomimetic composite scaffold was measured using the haemolysis ratio and platelet adhesion rate. Venous blood was collected from a healthy volunteer in trisodium citrate (9%) vials in accordance with ISO 10993–4: 2017 [37]. The haemolysis ratio was evaluated by measuring the relative amounts of haemoglobin released into the solution phase from the erythrocytes in whole blood exposed to the test materials [38].

Small pieces (1 cm^2^ × 0.1 cm) of PLA/GO/Sal-B/ASA dual drug-loaded biomimetic composite scaffolds were placed in tubes with 10 mL of normal saline and kept warm at 37 °C for 30 min. For haemolytic rate quantification, 0.2 mL of diluted anticoagulant blood (8 mL of fresh anticoagulant blood in 10 mL 0.9% normal saline) was added into the tubes to soak the composite scaffold at 37 °C for 60 min under gentle agitation. Additionally, 0.2 mL of diluted anticoagulant blood was added to tubes with normal saline and distilled water to serve as negative and positive controls, respectively. The control group was also subjected to constant temperature oscillation at 37 °C for 60 min. After the pre-determined culture time was completed, the immersion solution was centrifuged at 2000 rpm for 10 min, and the absorbance of the supernatant was determined at 540 nm via spectrophotometry. The final data from each group were obtained by averaging the values of three samples. The haemolytic rate was calculated using the following formula [39]:

(3) $$HR(\%)=\frac{X_{\mathrm{ts}}-X_{\mathrm{nc}}}{X_{\mathrm{pc}}-X_{\mathrm{nc}}}\times 100\%$$

where *X*_ts_ is the absorbency of the test scaffold, and *X*_nc_ and *X*_pc_ are the absorbance values of negative and positive controls, respectively.

The PLA/GO/Sal-B/ASA dual drug-loaded composite scaffolds (1 cm^2^ × 0.05 cm) were immersed in normal saline and incubated at 37 °C for 2 h to investigate their platelet adhesion. Fresh human anticoagulant blood was centrifuged at 3000 rpm for 20 min to obtain platelet-rich plasma (PRP). Normal saline was removed, and 0.2 mL of fresh PRP was added on the surface of the PLA/GO/Sal-B/ASA dual drug-loaded biomimetic composite scaffolds, which were maintained at 37 °C for 2 h. The scaffolds were then carefully rinsed with normal saline three times to remove the non-adherent platelets. Finally, the scaffolds were fixed to the platelets using 2.5 wt% glutaraldehyde at 4 °C for 1 h. The samples were rinsed with PBS twice and dehydrated by immersing them into a series of ethanol–deionized water solutions (50%, 60%, 80%, 90% and 100% [*v*/*v*]) for 30 min each. After dehydration, the samples were incubated at 4 °C for 24 h and vacuum freeze-dried for further use. Platelet adhesion was observed under SEM after gold coating.

### 2.10. Statistical Analysis

All in vitro experiments were performed in triplicate, and data were expressed as means ± standard deviation (SD). Statistical analysis was conducted using GraphPad Prism software (v. 7.0). *p*-values between two groups were determined using Student’s t-test. *p* < 0.0001 was considered statistically significant.

## 3. Results and Discussion

### 3.1. Morphology of PLA/GO/ASA/Sal-B Dual Drug-Loaded Biomimetic Composite Scaffolds

**Figure 2:**
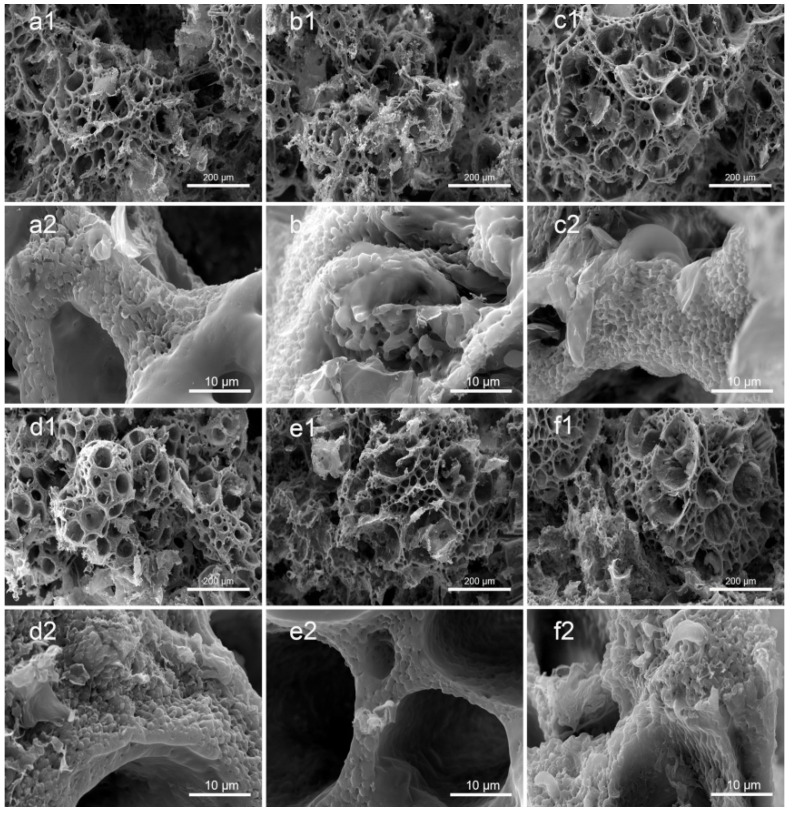
Morphology of the dual drug-loaded biomimetic composite scaffold: (**a1**,**a2**) S0A5; (**b1**,**b2**) S1A5; (**c1**,**c2**) S3A5; (**d1**,**d2**) S5A5; (**e1**,**e2**) S3A10 and (**f1**,**f2**) S3A15.

Figure 2a–d shows the morphology of the PLA/GO/Sal-B/ASA dual drug-loaded biomimetic composite scaffolds with a Sal-B content of 0, 1.0, 3.0 and 5.0 wt%, respectively, and a fixed ASA content of 5.0 wt%. Figure 2c,e,f show the morphology of the dual drug-loaded scaffolds with a fixed Sal-B content of 3 wt% and an ASA content of 5.0, 10.0 and 15.0 wt%, respectively. The GO and PLA content of the dual drug-loaded series of composite scaffolds were 2.0 wt% and 10.0 wt%, respectively. Figure 2 shows that the matrix structures of the scaffolds were porous spherulite structures, and powder and flake structures were uniformly dispersed on the surface of the scaffolds (Figure 2(a1–f1)). The PLA used in this paper is a semi-crystalline polymer, so PLA crystallizes out during phase separation. The crystalline morphology of polymers varies, including spherulite, lamellar crystal and fibrous crystal, among which spherulite is the most common crystal morphology. The formation of the spherulite porous structure may be due to the volatilization of the solvent, which was embedded as an impurity during the crystal growth [40]. Enlargement of the spherulite structures showed that the framework of the scaffolds was composed of dense fiber networks, and the fiber size was relatively coarse, up to 3–5 µm. The surface of the fiber showed a layer of coating. Therefore, the prepared dual drug-loaded composite scaffold is likely to have a biomimetic nanofiber structure after sustained release.

The mechanism of the formation of the nanofiber structure is not completely clear. Peter et al. reported that the crystallization of PLA may be a key factor in the formation of nanofiber structures [41,42]. Previous studies also showed that the formation of a gel during phase separation is key to the formation of nanofiber structures [43]. Indeed, in the preparation process of this scaffold, according to the previous preparation process of PLA/GO biomimetic composite scaffolds, the scaffold could not form a gel after being cured at 0 °C for 3 h, and the structure of the dual drug-loaded composite scaffold did not have a nanofiber network (data not shown). Therefore, the curing time of the scaffold was elongated during the preparation process, the scaffold was frozen at −40 °C until the polymer mixture formed a gel, and the nanofiber structure of the biomimetic extracellular matrix eventually formed. Previous studies have shown that the formation of PLA microcrystals act as a cross-linking agent for gel formation, promoting the formation of a gel and ultimately leading to the formation of nanofiber structures [43]. The simultaneous addition of two drugs delayed the formation of the gel, which may be because the addition of these drugs became a plasticizer of PLA and promoted the activity of the PLA molecular chain, making it difficult for microcrystals to stabilize. As the curing time increased, the solvent in the system was gradually frozen, thus increasing the viscosity of the polymer mixture system, gradually stabilizing the microcrystals and then forming a gel, making the nanofiber structure stable. Therefore, under controlled phase separation conditions, double drug-loading scaffolds can still form biomimetic nanofiber drug-loading scaffolds with a drug-loading capacity.

### 3.2. XRD and FTIR of PLA/GO/ASA/Sal-B Dual Drug-Loaded Biomimetic Composite Scaffolds

**Figure 3:**
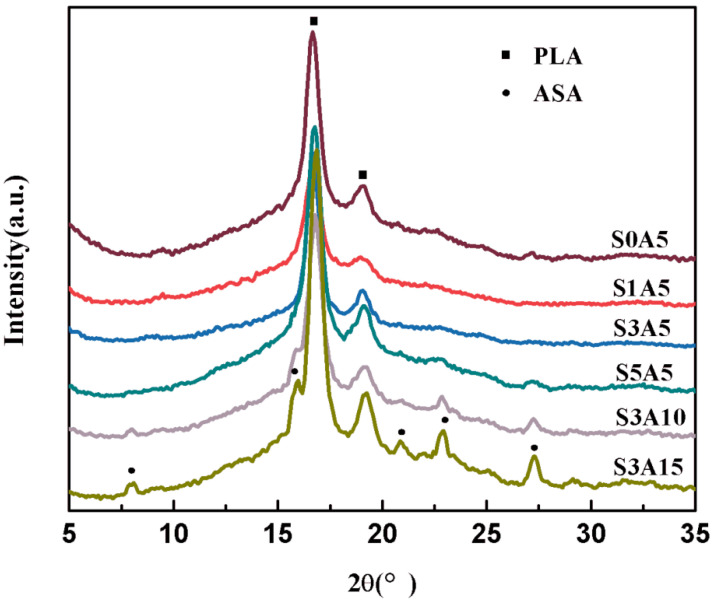
XRD of the double drug-loaded biomimetic composite scaffold.

Figure 3 illustrates the XRD patterns of PLA/GO/Sal-B/ASA biomimetic composite scaffolds. The XRD patterns do not observe the characteristic diffraction peak of Sal-B at 20°, possibly because of its fine and even dispersion. However, the intensity of the characteristic diffraction peaks of PLA at 17° and 19° first weakened and then strengthened with increasing Sal-B content. These results indicate that the addition of a small amount of Sal-B interferes with the crystallization of PLA and affects its crystallinity. The peak strength increases again as the content of Sal-B increases, which may be because Sal-B acts as a plasticizer, enhancing the movement of the PLA molecular chain, thus increasing the final crystallinity. By comparing the spectra of S3A5, S3A10, and S3A15, the characteristic diffraction peaks of ASA at 7°, 16°, 20.8°, 22.9°, 27.3° and 33° appear in the spectra when the ASA content increases to more than 10.0 wt%, and the intensity increases with the ASA content [44]. The XRD results showed that Sal-B and ASA were successfully compounded into the scaffold, and a small amount of Sal-B affected the crystallization of PLA but did not affect the final biomimetic structure (Figure 2).

**Figure 4:**
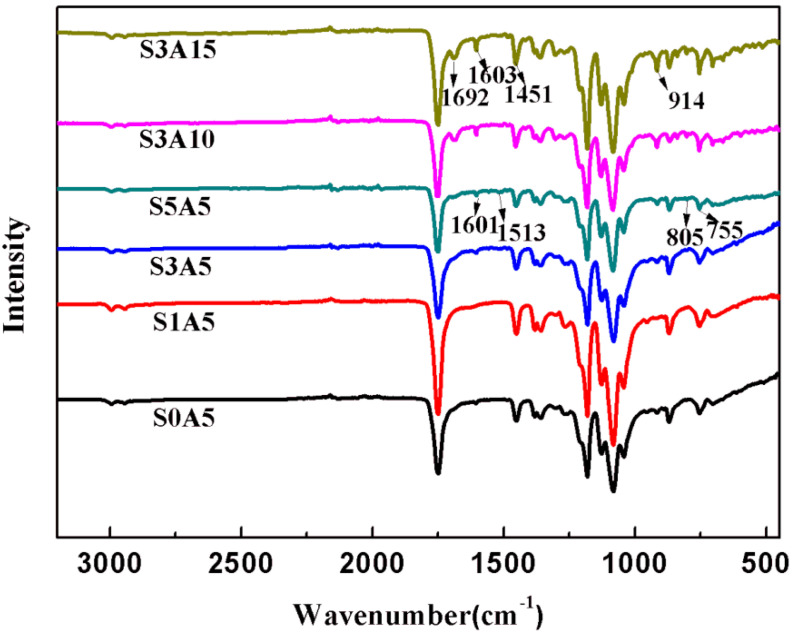
FTIR of the dual drug-loaded biomimetic composite scaffold.

Figure 4 shows the FTIR diagram of PLA/GO/Sal-B/ASA biomimetic composite scaffolds loaded with different Sal-B and ASA amounts. As shown in the figure, the FTIR curves of different Sal-B amounts (S0A5–S5A5) showed characteristic absorption peaks at 1601 cm^−1^, 1513 cm^−1^, 805 cm^−1^ and 755 cm^−1^ with increasing Sal-B content [45]. Among these, 1601 cm^−1^ is the characteristic absorption wavelength of phenolic acids [9]. Additionally, the FTIR curves of S3A5, S3A10 and S3A15 showed that the intensity of the out-of-plane bending vibration absorption peak of -OH in ASA near 912 cm^−1^ increased with increasing ASA content, and this is the characteristic absorption peak of ASA dissociation [46]. Increasing ASA also strengthened the stretching vibration characteristic peak (1692 cm^−1^) of the C=O dissociation linked to the benzene ring [46]. Meanwhile, the characteristic absorption peaks of PLA at 1751 cm^−1^, 1181 cm^−1^, 1081 cm^−1^ and 867 cm^−1^ appeared in all FTIR curves [34]. These results show that both Sal-B and ASA were successfully compounded into PLA/GO/Sal-B/ASA dual-loaded scaffolds.

### 3.3. Water Absorption of PLA/GO/ASA/Sal-B Dual Drug-Loaded Biomimetic Composite Scaffolds

**Figure 5:**
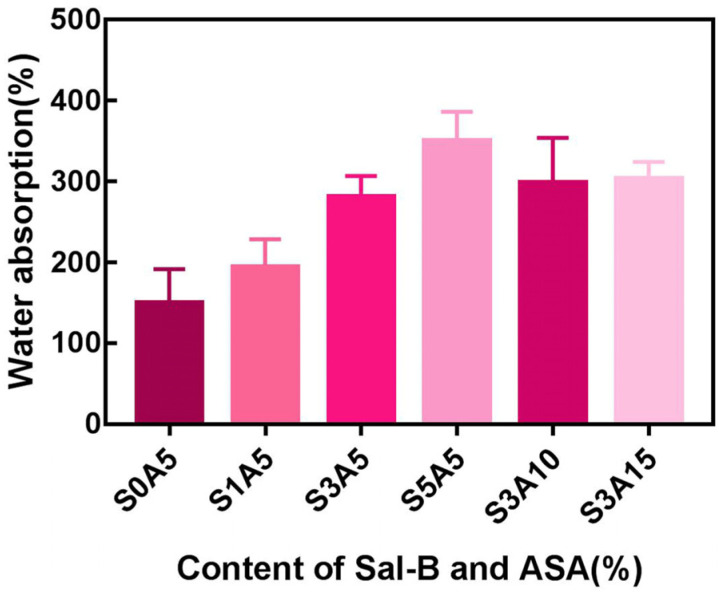
Water absorption of the dual drug-loaded biomimetic composite scaffold.

Water absorption is an effective method to characterize the hydrophilicity of scaffolds. As shown in Figure 5, when the ASA content remained unchanged, the water absorption rate of the PLA/GO/Sal-B/ASA dual drug-loaded composite scaffolds increased gradually with increasing Sal-B content. However, when the content of Sal-B was fixed, the water absorption of the composite scaffold changed little. This may be related to the water solubility of the two drugs themselves. Sal-B is a water-soluble phenolic acid, so the water absorption of the composite scaffold increases as the Sal-B content increases. However, the water solubility of ASA is not very good, so when the content of Sal-B is fixed, the water absorption of the dual drug-loaded scaffold does not change much. However, regardless of the proportion of the two drugs, the water absorption rate of the dual drug-loaded composite scaffolds was relatively high; that is, the prepared dual drug-loaded scaffolds had good hydrophilicity, which plays an important role in subsequent drug release, cell adhesion, and nutrient transport.

### 3.4. Porosity of PLA/GO/ASA/Sal-B Dual Drug-Loaded Biomimetic Composite Scaffolds

**Figure 6:**
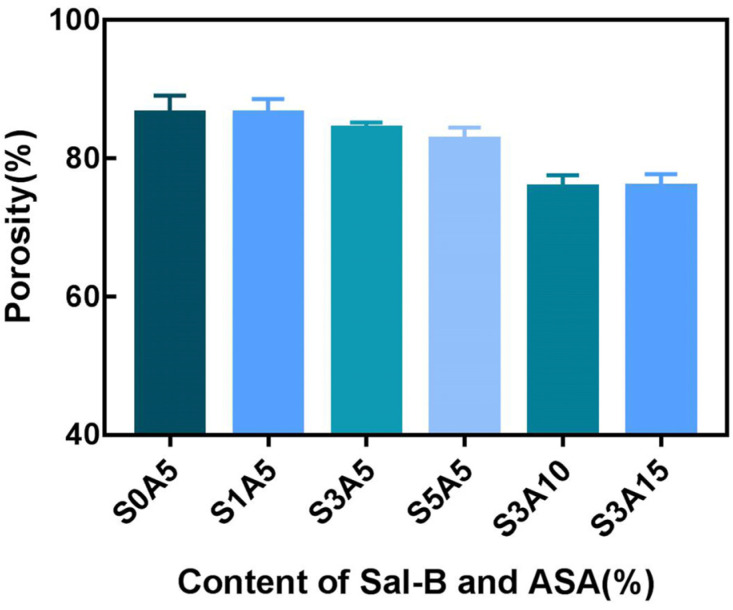
Porosity of dual drug-loaded biomimetic composite scaffold.

Figure 6 shows the porosity of the PLA/GO/Sal-B/ASA biomimetic composite scaffolds with different amounts of Sal-B and ASA. As shown in Figure 6, when the ASA content remained unchanged, the porosity of the PLA/GO/Sal-B/ASA dual drug-loaded composite scaffolds showed a downward trend with increasing Sal-B content, but the range was not significant, which may be because the amount of Sal-B was not large, so the results did not change significantly. When the amount of Sal-B remained unchanged, the porosity of the dual drug-loaded scaffold decreased with increasing ASA content, and when the ASA content was greater than 10 wt%, the porosity of the scaffold was lower than 80%, which was not conducive to the application of the scaffold as a bone repair material [47].

### 3.5. Haemocompatibility of PLA/GO/ASA/Sal-B Dual Drug-Loaded Biomimetic Composite Scaffolds

**Figure 7:**
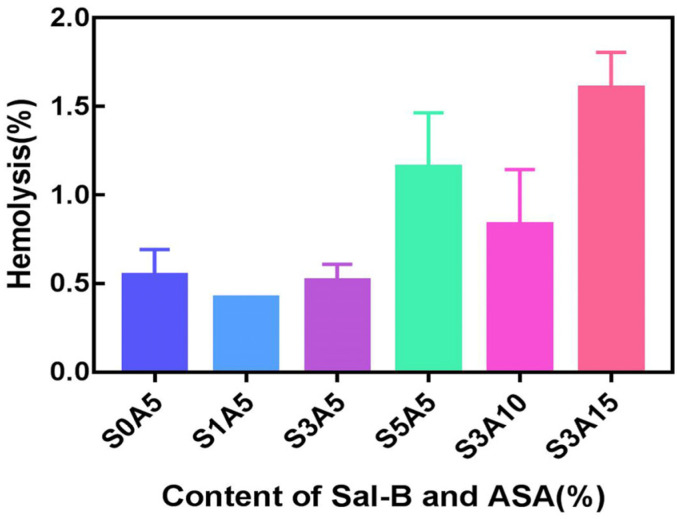
Haemolysis rate of the dual drug-loaded biomimetic composite scaffold.

Haemolysis tests were performed on PLA/GO/Sal-B/ASA dual drug-loaded scaffolds in two series to demonstrate the compatibility of the scaffold surface with human erythrocytes. Figure 7 shows that the haemolysis rates of all scaffolds were less than 2.0%. According to the regulations of the American Society for Testing and Materials (ASTM F756-00.2000) [48], the two series of dual drug-loaded scaffolds are non-haemolytic materials, and both have good blood compatibility. The figure also shows that when the ASA content remained unchanged, the addition of 1.0 wt% Sal-B provided the scaffold with the minimum haemolytic rate, and a subsequent increase in Sal-B content led to an increase in the haemolysis rate, which is contrary to the trend of Sal-B on the haemolysis rate in PLA/GO/Sal-B drug-loaded biomimetic composite scaffolds. This may be due to the synergistic effect of ASA and Sal-B on human erythrocytes. However, when the amount of Sal-B was fixed, the haemolysis rate increased with increasing ASA content, which was consistent with the haemolysis trend of PLA/GO/ASA drug-loaded scaffolds [34], indicating that the effect of Sal-B on haemolysis inhibition was better than that of ASA. However, how the two contribute to haemolysis together remains to be studied.

**Figure 8:**
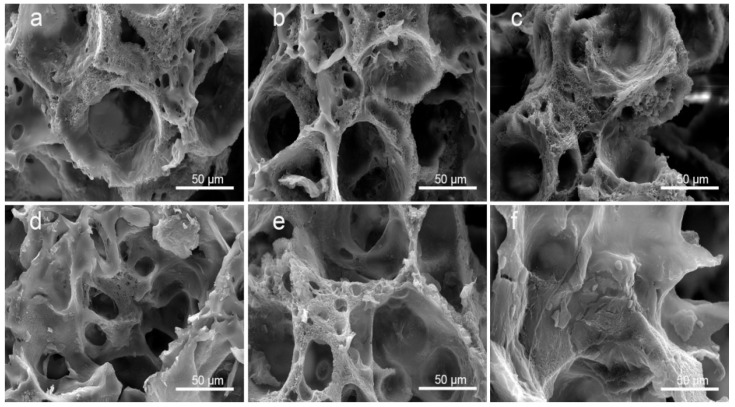
SEM of platelet adhesion for dual drug-loaded biomimetic composite scaffolds: (**a**) S0A5, (**b**) S1A5, (**c**) S3A5, (**d**) S5A5, (**e**) S3A10 and (**f**) S3A15.

Figure 8 shows the SEM diagram of platelet adhesion of the dual drug-loaded composite scaffolds with different amounts of Sal-B and ASA. The results show that platelet adhesion was not observed on the surface of the drug-loaded composite scaffolds. The surface properties, hydrophilicity and composition of these composite scaffolds may play a role in platelet adhesion [49]. Previous studies have shown that both Sal-B and ASA do not adhere to platelets, which may be due to the characteristics of these two drugs, both of which show good antiplatelet aggregation functions. ASA is an anticoagulant that has been shown to increase blood flow and decrease platelet activity. Lee [50] and Aslani [51] reported that a biodegradable nanofiber composite scaffold loaded with ASA effectively inhibited platelet adhesion. Ren et al. reported that Sal-B plays an important role in the cardiovascular field because of its inhibitory effect on platelet adhesion and aggregation [52]. Liu et al. showed that Sal-B had an antiplatelet effect, demonstrating that Sal-B inhibited human platelet activation by inhibiting PDE and antagonizing P2Y12 [49].

### 3.6. Cell Proliferation

**Figure 9:**
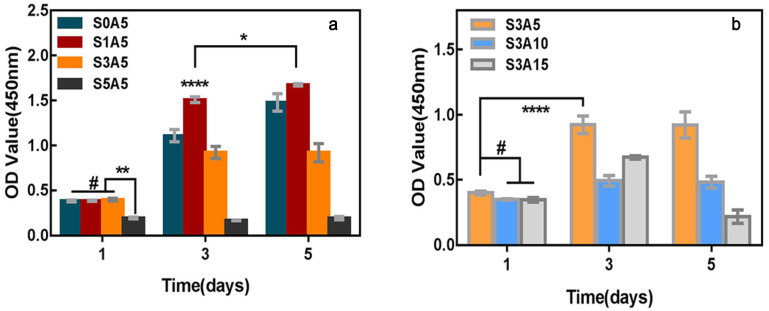
MC3T3-E1 cell proliferation on PLA/GO/Sal-B/ASA dual drug-loaded biomimetic composite scaffolds with different Sal-B content (**a**) and different ASA content (**b**) after 1, 3 and 5 days of culture (*n* = 3, mean ± SD, # *p* > 0.05, * *p* < 0.05, ** *p* < 0.01 and **** *p* < 0.0001).

Figure 9a shows the CCK-8 results of MC3T3-E1 cells cultured with biomimetic composite drug-loading scaffolds with different Sal-B amounts, while the ASA content was fixed at 5.0 wt%. As shown in the figure, MC3T3-E1 cells proliferated on all scaffolds, but the effect varied with the amount of Sal-B. The S0A5, S1A5 and S3A5 scaffolds showed no significant difference between the groups after 1 d of culture (*p* > 0.05), but the proliferation of the scaffolds in the three groups was significantly higher than that in S5A5 (*p* < 0.01). S0A5, S1A5 and S3A5 scaffolds also significantly proliferated after 3 and 5 d of culture (*p* < 0.0001), while S5A5 had no clear proliferation at 3 d and 5 d. Meanwhile, the cell proliferation of S1A5 was superior to that of S0A5, indicating that a small amount of Sal-B was beneficial to the proliferation of MC3T3-E1 cells.

Figure 9b shows the CCK-8 results of MC3T3-E1 cells cultured with biomimetic composite drug-loaded scaffolds with different ASA amounts, while the Sal-B content was fixed at 3.0 wt%. As shown in the figure, MC3T3-E1 cells proliferated on all scaffolds, but the effect varied with ASA amount. After being cultured for 3 d, all scaffolds showed higher cell proliferation than that after culturing for 1 d (*p* < 0.0001). However, after the fifth day of culture, the cell proliferation of the S3A15 group decreased significantly (*p* < 0.0001), and there was no significant difference between the other two groups (*p* > 0.05). These results indicate that a high concentration of ASA (15.0 wt%) had an inhibitory effect on MC3T3-E1 cells. However, compared with previous studies of PLA/GO/ASA [34], the presence of Sal-B prevented the inhibition of ASA on cell proliferation in the first 3 d of culture; that is, the addition of Sal-B delayed the inhibition of a high concentration of ASA on cells, which may represent the synergistic effect of the two drugs on cells. Other research has also shown that Sal-B promoted the proliferation of MC3T3-E1 cells [53].

### 3.7. Alkaline Phosphatase Activity

**Figure 10:**
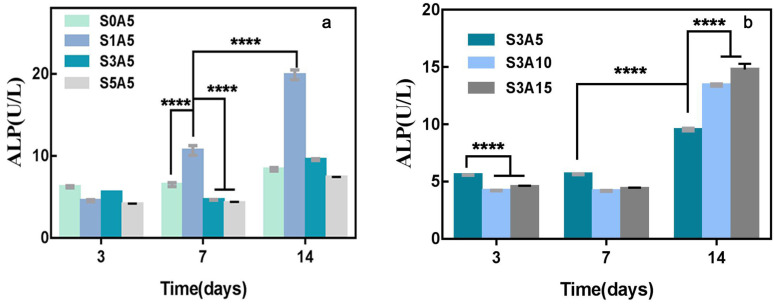
ALP activity of MC3T3-E1 cells cultured with dual drug-loaded biomimetic composite scaffolds with different Sal-B content (**a**) and different ASA content (**b**). (*n* = 3, mean ± SD, **** *p* < 0.0001).

ALP is one of the first functional biomolecules expressed in the calcification process, which is suggesting the crucial role of this enzyme in the early stages of mineralization [54]. Therefore, the measurement of scaffold-induced ALP differentiation can be used to characterize the osteogenic properties of drug-loaded scaffolds. To evaluate the effect of co-loaded Sal-B and ASA as bone tissue engineering scaffolds, the activity of ALP after the scaffolds were cultured in vitro was measured. Figure 10a shows that the ALP activity of the composite scaffolds was affected by different Sal-B amounts when the ASA content was fixed at 5.0 wt%. The results show that the ALP activity of the scaffold without Sal-B (S0A5) was significantly increased after 14 d of culture compared with 7 d (*p* < 0.0001), and there was no significant difference between day 3 and day 7 (*p* > 0.05). The ALP activity of S3A5 and S5A5 was also significantly increased after 14 d of culture, while the ALP activity of S1A5 was significantly increased at all three measured time points (*p* < 0.0001). These results show that the presence of a small amount of Sal-B (1 wt%) increased the ALP activity of the dual drug-loaded scaffolds quickly and effectively; that is, it quickly induced bone differentiation. Figure 10b shows the ALP measurement results of composite drug-loading stents with different ASA amounts while the amount of Sal-B was fixed. The figure shows that there was no significant difference in the ALP activity of the same sample at different time points when the three samples were cultured for 3 d and 7 d, but the samples in the S3A5 group had the highest ALP activity compared to the other two groups (*p* < 0.0001). However, with an increase in culture time, the ALP level of the three samples was significantly enhanced on the 14th day compared with the previous two time points, and S3A10 and S3A15 had higher ALP activity. These results indicate that an increase in ASA in the dual drug-loaded scaffolds is beneficial to the expression of ALP activity in a dose-dependent manner.

### 3.8. The Sustained Release of the Two Drugs

**Figure 11:**
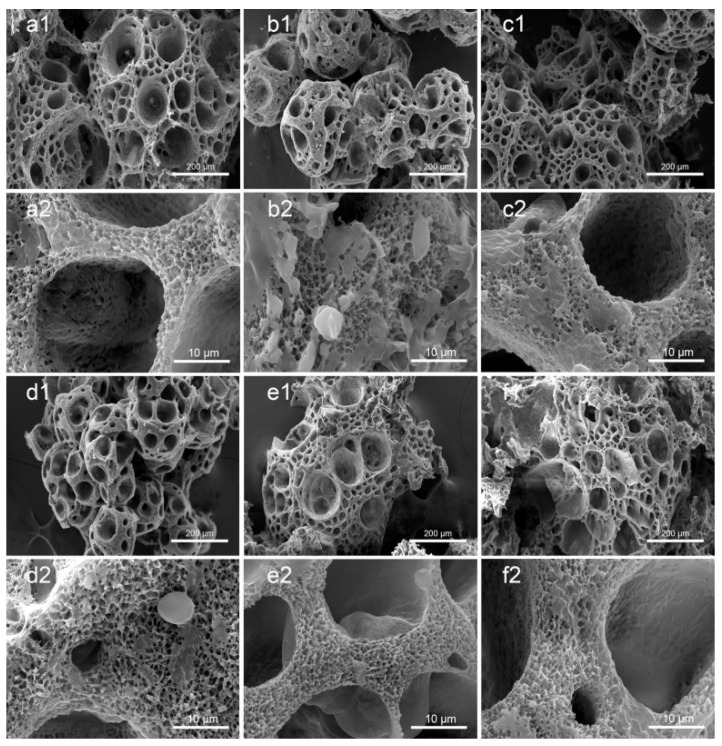
Morphology of dual drug-loaded biomimetic composite scaffold after sustained release for 9 days: (**a1**,**a2**) S0A5, (**b1**,**b2**) S1A5, (**c1**,**c2**) S3A5, (**d1**,**d2**) S5A5, (**e1**,**e2**) S3A10 and (**f1**,**f2**) S3A15.

Figure 11 shows the SEM figure of the PLA/GO/Sal-B/ASA biomimetic composite scaffolds with different amounts of Sal-B and ASA after soaking in PBS buffer solution for 9 d. Compared with Figure 2, the drug-loaded scaffold after sustained release was composed of a uniform nanofiber network structure, and the fiber diameter was much smaller than before sustained release. The fiber diameter was from the micrometer scale to the nanometer scale, and the fiber network was observed. Although the fiber diameter of the PLA/GO/Sal-B/ASA dual drug-loaded scaffolds was composed of micro and nanoscale fibers before the sustained release of the drugs, the fiber diameter of the scaffolds decreased significantly after their sustained release. The results showed that the surface of the fiber skeleton of the drug-loaded biomimetic scaffolds was covered with Sal-B or ASA. When the scaffold was immersed in PBS, Sal-B, and ASA, the surface of the scaffolds dissolved or diffused into the solution, resulting in a significant reduction in fiber diameter. The SEM diagram after sustained release showed that the PLA/GO/Sal-B/ASA dual drug-loaded biomimetic composite scaffolds possessed the micro-nano fiber structure of the biomimetic extracellular matrix after the sustained release of the drugs, and it still showed the advantages of a biomimetic structure, which plays an important role in subsequent scaffold-induced cell adhesion, proliferation, and other functions.

**Figure 12:**
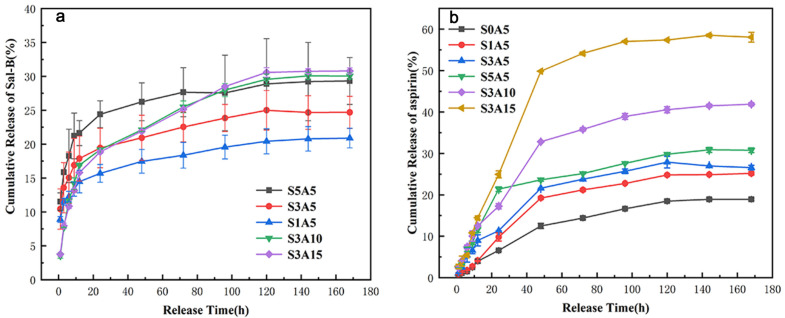
Cumulative release of dual drug-loaded biomimetic nanofibrous scaffolds: Sal-B (**a**) and ASA (**b**).

Figure 12a shows the curve of the cumulative release amount of Sal-B in the dual drug-loaded scaffolds. While the ASA content is fixed, all scaffolds demonstrated similar cumulative release curves, which can be divided into three stages: initial detonation (12 h), sustained release (12–120 h), and equilibrium (120–168 h). As shown in the figure, the release of Sal-B in S1A5, S3A5, and S5A5 showed the same trend. With an increase in Sal-B content, the cumulative release gradually increased, and the cumulative release at equilibrium was 20.90%, 24.70% and 29.32%, respectively. This was significantly lower than the cumulative release of the drug-loaded scaffolds without ASA at the same time points (the cumulative release of Sal-B in the PLA/GO/Sal-B series at 168 h was 44.54%, 65.58% and 86.36%, respectively), indicating that the addition of ASA slowed the release of Sal-B. At the same time, when the ASA content was greater than 5.0 wt%, the release curve of Sal-B in the S3A10 and S3A15 groups was slightly different from that of the other three groups, mainly reflecting the smaller amount of Sal-B cumulatively released in the initial stage, which was 16.92% and 15.81%, respectively, at 12 h. However, the release amount of the S3A5 group was 17.88%; at the initial stage, with an increase in ASA content, the release amount of Sal-B decreased. However, in the second stage, the S3A10 and S3A15 groups had relatively large release rates, and in the final equilibrium stage, they had even larger cumulative release amounts of 30.05% and 30.80%, respectively, which were still smaller than the cumulative release amounts of single-loaded Sal-B without ASA. These results indicate that the addition of ASA contributes to the slow release of Sal-B. This may be explained by the poor water solubility of ASA, and its addition hinders the diffusion of Sal-B in PBS solution.

Figure 12b shows the curve of the cumulative release of ASA in the dual drug-loaded scaffolds. When the ASA content was 5.0 wt%, the cumulative release of ASA increased with increasing Sal-B content, but the cumulative release of the S1A5, S3A5, and S5A5 scaffolds showed little change, which may be related to the solubility of Sal-B. The addition of the hydrophilic Sal-B promoted the wetting of PBS solution on the surface and inside the scaffold and increased the diffusion of ASA, thus increasing the release amount of ASA in the initial stage. At the same time, when the content of Sal-B is unchanged, the cumulative release of ASA increases gradually with the ASA content.

Bone repair and tissue reconstruction are complex processes, including (1) the initial inflammatory stage, (2) the proliferation stage of tissue regeneration to fill the wound and (3) the maturation and remodeling stage to restore bone function. Therefore, an ideal bone scaffold should contain different biomolecules involved in each stage of bone repair. ASA is widely used as an anti-inflammatory drug [12,13], and the inflammatory reaction also affects the healing of bone tissues in the later stages [9]. Sal-B is a small bioactive molecule that is beneficial to bone induction and vascularization [9]. As for the application of drug-loaded bone tissue engineering scaffolds in bone repair, we hypothesized that inflammation may occur in the early stage of transplantation, requiring more and faster ASA release; on the other hand, slow and sustained release of Sal-B is required to promote angiogenesis and re-epithelialization during bone healing and repair [9]. Therefore, dual drug-loaded biomimetic composite scaffolds can promote the faster release of ASA, while slowing down and sustaining the release of Sal-B. In this way, the co-loading of various biomolecules with different dissolution properties can achieve phased release in different bone healing stages.

**Figure 13:**
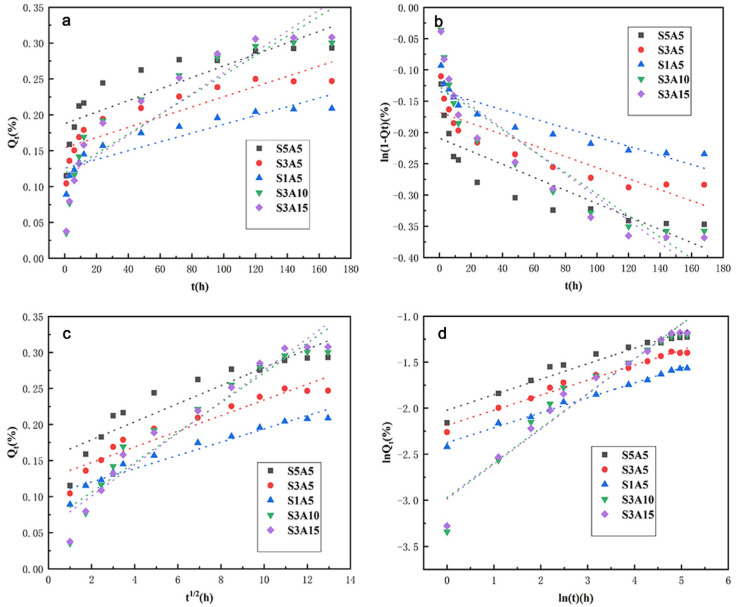
Drug release kinetic model plots of Sal-B from dual drug-loaded biomimetic composite scaffolds: Zero-order (**a**), First-order (**b**), Higuchi (**c**) and Korsmeyer–Peppas (**d**).

**Figure 14:**
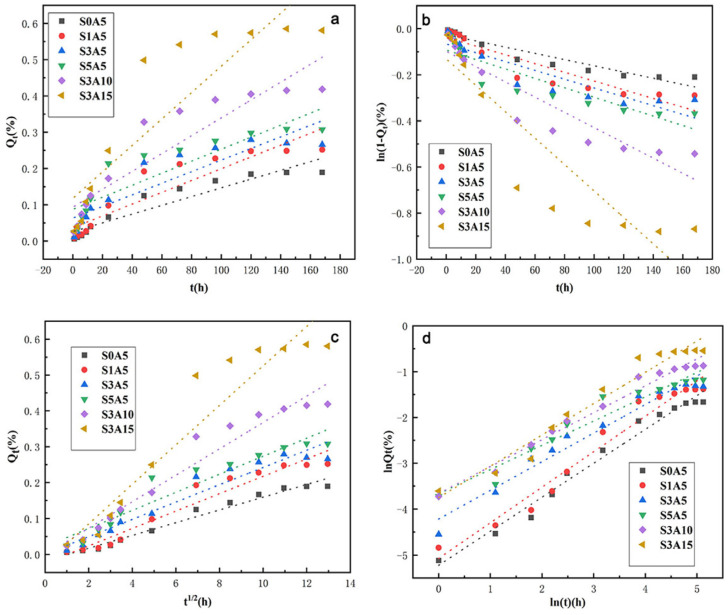
Drug release kinetic model plots of ASA from dual drug-loaded biomimetic composite scaffolds: Zero-order (**a**), First-order (**b**), Higuchi (**c**) and Korsmeyer–Peppas (**d**).

To better study the release mechanism of Sal-B and ASA, in vitro release data of Sal-B and ASA were linearly fitted by four mathematical dynamics models: the zero-order kinetics equation, the first-order kinetics equation, the Higuchi equation and the Korsmeyer–Peppas equation. Corresponding equations and correlation coefficients of these models are given in Table 2 and Table 3, respectively. Meanwhile, linear fitting curves of the four dynamic models of Sal-B and ASA are presented in Figure 13 and Figure 14, respectively. According to the comparison of Table 2, for the linear fitting of the four kinetic models of Sal-B in the dual drug-loaded scaffolds, the correlation coefficient R^2^ of the Korsmeyer–Peppas model for all drug-loading scaffolds was the largest, followed by the Higuchi model, and the zero-order model had the smallest R^2^ value. These results indicate that the release of Sal-B from the dual drug-loaded scaffolds was most consistent with the Korsmeyer–Peppas model.

The Korsmeyer–Peppas model was developed specifically for the drug delivery of polymer carriers [55,56,57]. In this model, the release mechanism is determined by the function of the relationship between the release index *n* and time, and the formula is expressed as follows:

(4) $$\ln Q_{t}=n\ln t+\ln k_{\mathrm{KP}}$$

where *Q*_t_ is the cumulative drug release amount of the scaffold at time *t*, *k*_KP_ is a kinetic constant related to the nature of the drug delivery system and *n* is the release index, which is a release mechanism index related to the carrier form. Different *n* values represent different release mechanisms [55], and the relationship between specific release mechanisms and carrier morphology is shown in Table 4. Given the scaffold morphology adopted in this study, membrane morphology was chosen as a reference. Therefore, when *n* is lower than 0.5, drug release follows Fick diffusion (controlled by diffusion), while when *n* is equal to 1.0, drug release is mainly according to swelling/dissolution. A value of *n* between 0.5 and 1.0 represents anomalous transport, which is characterized by a combination of the two phenomena. However, the Korsmeyer–Peppas model only applies to *Q*_t_ < 0.6 in the sustained-release system [55,58]. The curve was plotted by ln*Q*_t_ against lnt, where the slope was *n*, and ln*k*_KP_ was the logarithm of the rate constant. The correlation coefficient R^2^ was obtained by fitting the curve (as shown in Table 2). As can be seen in Table 2, the release index n of the series of drug-loading scaffolds in this model was less than 0.5. According to Table 4, the release mechanism of Sal-B in the dual-loaded scaffolds therefore follows the Fick diffusion mechanism; that is, diffusion plays a major role in the drug release process [55].

## 4. Conclusions

PLA/GO/Sal-B/ASA dual drug-loaded biomimetic composite scaffolds were prepared by thermally induced phase separation. The effects of different amounts of Sal-B and ASA on the structure and physicochemical properties of the biomimetic nanofiber scaffolds were investigated. The addition of the two drugs together delayed the gel formation of the composite system, but the biomimetic nanofiber structure was still obtained by extending the gelling time. XRD and FTIR results showed that both Sal-B and ASA could effectively compound onto the scaffold. The addition of Sal-B was beneficial for increasing the hydrophilicity of the scaffold, but ASA was not. In addition, an increase in ASA caused the porosity of the scaffold to decrease to less than 80%. Both dual drug-loaded scaffolds had good blood compatibility, and Sal-B and ASA synergically promoted MC3T3-E1 cell proliferation and enhanced ALP activity. Sustained-release experiments of the two drugs showed that the presence of ASA slowed the cumulative release of Sal-B, while Sal-B promoted the release of ASA. Kinetic modeling showed that the release models of both Sal-B and ASA were consistent with the Korsmeyer–Peppas model, but Sal-B was consistent with the Fick diffusion mechanism, and ASA follows the release mechanism of Fick diffusion and carrier swelling/dissolution.

## Figures and Tables

**Table 1:** The composition ratio of composite scaffolds with different drug contents.

	Name	S0A5	S1A5	S3A5	S5A5	S3A10	S3A15
Composition(%)							
PLA		10.0	10.0	10.0	10.0	10.0	10.0
GO		2.0	2.0	2.0	2.0	2.0	2.0
Sal-B		0.0	1.0	3.0	5.0	3.0	3.0
ASA		5.0	5.0	5.0	5.0	10.0	15.0

The PLA content is a percentage relative to the solvent; The content of GO, Sal-B and ASA is relative to the mass percentage of PLA.

**Table 2:** Fitting parameters of drug release kinetics of Sal-B from the dual drug-loaded biomimetic composite scaffolds.

Model		Zero-Order		First-Order		Higuchi		Korsmeyer–Peppas		
	Parameters	R^2^	k_0_	R^2^	k_1_	R^2^	k_H_	R^2^	*n*	k_kp_
Sample										
S1A5		0.6513	8.03 × 10^−4^	0.6791	1.04 × 10^−3^	0.6700	3.19 × 10^−4^	0.9396	0.1686	0.1324
S3A5		0.7500	7.11 × 10^−4^	0.7695	8.85 × 10^−4^	0.7631	2.74 × 10^−4^	0.9795	0.1637	0.1123
S5A5		0.8075	6.12 × 10^−4^	0.8224	7.32 × 10^−4^	0.8175	2.30 × 10^−4^	0.9923	0.1627	0.0929
S3A10		0.7789	1.37 × 10^−3^	0.8134	1.72 × 10^−3^	0.8023	5.30 × 10^−4^	0.9263	0.3770	0.0512
S3A15		0.8194	1.45 × 10^−3^	0.8501	1.83 × 10^−3^	0.8402	5.65 × 10^−4^	0.9557	0.3802	0.0504

**Table 3:** Fitting parameters of drug release kinetics of ASA from the dual drug-loaded biomimetic composite scaffolds.

Model		Zero-Order		First-Order		Higuchi		Korsmeyer–Peppas		
	Parameters	R^2^	k_0_	R^2^	k_1_	R^2^	k_H_	R^2^	*n*	k_KP_
Sample										
S0A5		0.8887	1.21 × 10^−3^	0.9002	1.35 × 10^−3^	0.8964	4.33 × 10^−4^	0.9790	0.7402	0.0054
S1A5		0.8345	1.63 × 10^−3^	0.8500	1.89 × 10^−3^	0.8449	6.00 × 10^−4^	0.9623	0.7744	0.0063
S3A5		0.8058	1.60 × 10^−3^	0.8219	1.91 × 10^−3^	0.8167	6.00 × 10^−4^	0.9598	0.6249	0.0147
S5A5		0.7742	1.66 × 10^−3^	0.8044	2.04 × 10^−3^	0.7944	6.35 × 10^−4^	0.9412	0.5299	0.0255
S3A10		0.8283	2.46 × 10^−3^	0.8582	3.31 × 10^−3^	0.8485	9.97 × 10^−4^	0.9778	0.5882	0.0257
S3A15		0.7785	3.64 × 10^−3^	0.8156	5.73 × 10^−3^	0.8035	1.63 × 10^−3^	0.9557	0.6837	0.0235

**Table 4:** Relationship between the release index and the shape of the drug release mechanism or the drug release system in the Korsmeyer–Peppas model [55,58].

Drug Release Mechanism	Release Index (*n*)		
	Ball	Cylinder	Film
Fickian diffusion	0.43	0.45	0.50
Non-fick diffusion (Random diffusion)	0.43 < *n* < 0.85	0.45 < *n* < 0.89	0.50 < *n* < 1.00
Swelling/dissolution of polymer	0.85	0.89	1.00

## Data Availability

Not applicable.
